# Multiple goals and time constraints: perceived impact on physicians' performance of evidence-based behaviours

**DOI:** 10.1186/1748-5908-4-77

**Published:** 2009-11-26

**Authors:** Justin Presseau, Falko F Sniehotta, Jillian J Francis, Neil C Campbell

**Affiliations:** 1 Health Psychology, School of Psychology, William Guild Building, University of Aberdeen, Aberdeen, UK; 2 Health Services Research Unit, University of Aberdeen, Third Floor Health Sciences Building, Foresterhill, Aberdeen, UK; 3 Centre of Academic Primary Care, University of Aberdeen, Westburn Road, Aberdeen, UK

## Abstract

**Background:**

Behavioural approaches to knowledge translation inform interventions to improve healthcare. However, such approaches often focus on a single behaviour without considering that health professionals perform multiple behaviours in pursuit of multiple goals in a given clinical context. In resource-limited consultations, performing these other goal-directed behaviours may influence optimal performance of a particular evidence-based behaviour. This study aimed to investigate whether a multiple goal-directed behaviour perspective might inform implementation research beyond single-behaviour approaches.

**Methods:**

We conducted theory-based semi-structured interviews with 12 general medical practitioners (GPs) in Scotland on their views regarding two focal clinical behaviours--providing physical activity (PA) advice and prescribing to reduce blood pressure (BP) to <140/80 mmHg--in consultations with patients with diabetes and persistent hypertension. Theory-based constructs investigated were: intention and control beliefs from the theory of planned behaviour, and perceived interfering and facilitating influence of other goal-directed behaviours performed in a diabetes consultation. We coded interview content into pre-specified theory-based constructs and organised codes into themes within each construct using thematic analysis.

**Results:**

Most GPs reported strong intention to prescribe to reduce BP but expressed reasons why they would not. Intention to provide PA advice was variable. Most GPs reported that time constraints and patient preference detrimentally affected their control over providing PA advice and prescribing to reduce BP, respectively. Most GPs perceived many of their other goal-directed behaviours as interfering with providing PA advice, while fewer GPs reported goal-directed behaviours that interfere with prescribing to reduce BP. Providing PA advice and prescribing to reduce BP were perceived to be facilitated by similar diabetes-related behaviours (*e.g.*, discussing cholesterol). While providing PA advice was perceived to be mainly facilitated by providing other lifestyle-related clinical advice (*e.g.*, talking about weight), BP prescribing was reported as facilitated by pursuing ongoing standard consultation-related goals (*e.g.*, clearly structuring the consultation).

**Conclusion:**

GPs readily relate their other goal-directed behaviours with having a facilitating and interfering influence on their performance of particular evidence-based behaviours. This may have implications for advancing the theoretical development of behavioural approaches to implementation research beyond single-behaviour models.

## Background

Translation of research evidence into clinical practice remains a challenge [1,2]. The behavioural sciences provide a number of well-developed, operationalised, and tested models of human behaviour that generalise across contexts that can inform implementation research [3]. Among models with the best predictive utility is the Theory of Planned Behaviour (TPB) [4]. Applied to a healthcare professional context, the TPB has been used to predict behaviour [5], evaluate change [6], develop behaviour change interventions [3], and as a framework for qualitative investigation [7]. A core assumption of the TPB is that the two most important determinants of whether a health professional will perform any particular behaviour are how strongly they intend to (behavioural intention) and whether they feel they can (*i.e.*, their perceived behavioural control). The model also specifies predictors whose effect on behaviour is mediated by the health professional's intention: what they think about the consequences of performing the behaviour (their attitude), their perception of other influential people's views about them performing it (their subjective norms), and, again, their perceived behavioural control. Underlying each of these three constructs are associated specific beliefs: behavioural (about the outcome of performing the behaviour), normative (about how important other people want them to act), and control beliefs (about factors that make it difficult or easy to perform the behaviour). Reviews of predictive prospective studies suggest that this model accounts relatively well for the variation in healthcare professional behaviour [5,8]. However, the model is not without its critics [9,10], and further theoretical development to inform implementation efforts seems warranted. For instance, there is a recognised need for further development of behavioural theories to better understand and promote health professionals' efficient uptake of guideline recommendations [1].

As with most quality improvement research, most (though not all [11] [Presseau J, Sniehotta FF, Francis JJ, Gebhardt WA: With a little help from my goals: Integrating intergoal facilitation with the theory of planned behaviour to predict physical activity, Submitted]) applications of the TPB isolate behaviours from the wider context of multiple behaviours and multiple goals pursued. To the best of our knowledge, none of the studies in systematic reviews of tests of social cognition models with health professionals [5,8] considered whether performing multiple goal-directed behaviours was perceived to influence a focal behaviour of interest. It seems unlikely that the performance of one goal-directed behaviour is isolated from the performance of another, particularly in busy clinical settings. This study therefore aimed to explore whether and to what extent GPs attribute their performance of a particular evidence-based behaviour to being influenced by other goal-directed behaviours they perform in a consultation.

### Interference and facilitation between healthcare delivery goal-directed behaviours

Competing demands may affect the delivery of evidence-based diabetes healthcare [12]. For instance, lack of time due to competing demands is a frequently identified barrier to implementing guideline recommendations [13,14]. Duration of consultations with GPs in the UK is limited to an average of 9.4 minutes [15]. This constraint might result in a GP wanting and needing to perform a number of goal-directed behaviours in a consultation, but being unable to perform them all. Sources of competing demands in clinical consultations often include patient, physician, and contextual factors [16]. Each of these may lead the GP to perform a behaviour in order to pursue a particular goal. For instance, elements on the patient's agenda (*e.g.*, 'get advice for weight loss') can provide competing demands by first being perceived by the GP, and then generating additional goal-directed behaviours for the GP (*e.g.*, 'give weight loss advice') to be performed during the consultation. Furthermore, GPs have their own agenda for the consultation involving them performing many goal-directed behaviours. Perceived competing demands can thus be viewed as the behaviours performed by the GP to pursue the goals for the consultation--their goal-directed behaviours--informed by what they want to and/or need to do based on contextual and patient factors. For instance, during a diabetes consultation the GP may measure blood pressure (BP), increase dosage of ACE-Inhibitor to reduce BP, prescribe a statin, measure foot pulses, provide advice on diet and exercise, discuss risks and also respond to issues that the patient brings up, and try to finish on time, amongst others. GPs' management of diabetes in a clinical consultation can therefore be conceptualised as a system of goal-directed behaviours that they perform to provide optimal patient care, which all compete for the limited resources available.

Limited resources lead to three potentially overlapping relationships between goal-directed behaviours [17]. Pursuing one goal may: interfere with pursuing another, either by accounting for time available or due to an incompatibility (*e.g.*, checking lipids and prescribing statins in response to test results are incompatible goals for a particular consultation because blood tests are not instantaneous); facilitate pursuing another, either instrumentally (*e.g.*, providing dietary advice for weight loss can lead to providing exercise advice) or due to overlapping means (*e.g.*, prescribing an ACE-inhibitor pursues the goals of achieving a contract target and lowering BP); or be independent of pursuing another (which is less likely in resource-constrained settings). Goal interference has been related to performance in professional contexts, including call-centres [18] and with university academics [19]. Some research has investigated the effect of goal interference on performance of health behaviours such as exercise, though this effect is not as clear [17,20]. Goal facilitation has received comparatively less research attention, though a prospective correlational study found that facilitating goals predicted variance in health behaviour [17]. This effect has been subsequently shown to be partially mediated by the TPB, indicating that perceived goal facilitation has both a direct and indirect effect on health behaviour [Presseau J, Sniehotta FF, Francis JJ, Gebhardt WA: With a little help from my goals: Integrating intergoal facilitation with the theory of planned behaviour to predict physical activity, Submitted]. The effect of perceived goal interference and facilitation may be increasingly relevant to more constrained settings such as clinical consultations. Tools, such as personal projects analysis [21], provide a replicable methodology for eliciting personally salient multiple goal-directed behaviours and assessing their perceived influence on performance of a particular goal-directed behaviour in a particular context [22]. Incorporating the role of GPs' competing goal-directed behaviours in a diabetes consultation is a new approach which may inform single-behaviour operationalisations of behavioural models such as the TPB used to investigate health professional behaviour.

### Physical activity and BP control in the diabetes consultation

Tight BP control and physical activity can reduce the risk of developing diabetes-related complications [23,24]. However, many people with diabetes do not meet recommended BP and physical activity levels. In Scotland, 74% of women and 58% of men with type 2 diabetes engage in less than 30 minutes of moderate to vigorous physical activity per week, compared to 41% of women and 36% of men without type 2 diabetes [25]. Primary care physicians are recognised as being at the front line of diabetes management [26]. The role of the GP has been defined to include 'promoting health, preventing disease, and providing cure, care, or palliation. This is done either directly or through the services of others according to health needs and the resources available within the community they serve, and assisting patients where necessary in accessing these services. [27]'However patient surveys found that only one-half of patients with diabetes received exercise advice in their last visit to the GP [28], and three-quarters reported having ever received exercise advice from a healthcare professional [29]. In the UK, an incentive structure is built into the contract of GPs that remunerates for achieving predefined quality targets [30], known as Quality and Outcomes Framework (QOF) points. For example, for the management of diabetes, one of the targets (DM12) currently remunerates GPs when up to 60% of their patients with diabetes achieve a BP of ≤145/85 mmHg at their last reading. Notably, this target level is higher than the current UK and Scottish guideline recommendation of <140/80 mmHg [31,32]. QOF data collected in primary care practices in the north-east of Scotland showed that a mean of 77.8% (standard deviation 7.7%) of people with diabetes achieved a BP of ≤145/85 mmHg [33]. However, between-practice variation ranged from 59.5% to 100% of patients. Thus, despite evidence-based guideline recommendations detailing effective pharmacological means of reducing BP to evidenced targets [31,32,34] and providing physical activity advice in primary care [35,36], implementation remains suboptimal. Better implementation of the evidence in these guideline recommendations could have important implications for risk reduction.

Drawing upon existing theory and methods from the behavioural sciences, this study represents a preliminary stage in a series of studies aiming to investigate how competing goal-directed behaviours influence health professionals' evidence-based motivation and action.

## Methods

### Sampling and recruitment procedures

We recruited a purposive heterogeneous sample of 12 GPs from ten practices in NHS Grampian (Scotland) to represent variation in gender, age, and rural/urban practice. Purposive heterogeneity sampling was used so that a variety of views could be studied. We targeted clinical colleagues of one of the authors (NCC). Fourteen GPs were informally contacted via email; twelve indicated their interest in participating and were subsequently formally invited via email or telephone within one week of the initial approach to arrange a time and location for being interviewed. Pragmatic sample size considerations were made on the basis of advice from Guest, Bunce, and Johnson, who found that they developed 92% of codes within the first 12 (of 60) interviews conducted [37].

### Data collection procedure

Semi-structured one-to-one interviews investigated factors that GP's perceived facilitate and hinder their performance of two particular behaviours within the diabetes consultation they are most involved in: provision of physical activity advice and prescription of anti-hypertensive medication to those with persistent high BP to control it to evidence-based guideline levels of <140/80 mmHg. Interviews were preferred over other methods as they provided the best fit with the theory-development research questions, allowing us to prompt participants for further elaboration. The interview topic guide was piloted with one GP, and amended subsequent to piloting and throughout the study to maximise content and feasibility within the target time (30 minutes; see Additional File 1 for final topic guide). Interviews lasted on average 31 minutes (range = 21 to 53 minutes), and all (except one phone interview) were conducted face-to-face either in an office at each general practice or else at a pre-arranged alternative location if requested. Upon obtaining signed consent from participating GPs, interviews were digitally recorded. All interviews were conducted by JP from 19 March to 30 July 2008.

### Analysis

Interviews were transcribed verbatim and then content analysed by JP using N-Vivo 7. We defined a coding scheme *a priori *based on the theory-based constructs of interest (*i.e.*, control beliefs, intention, goal facilitation, and goal interference). Self-reported past behaviour was included to identify the extent to which these behaviours were performed. Construct definitions used for coding followed advice and examples from the literature [17,38,39]. Content relating to each theory-based construct was identified and coded from each interview by JP, then organised into representative themes for each theory-based construct using thematic analysis [40]. Analysis of the content within each theme was reviewed by a practising GP (NCC) who independently organised the coded content for each construct into representative themes. Disagreements were resolved by discussion. Coded content for perceived intergoal facilitation and interference were further analysed along a temporal dimension to investigate the relative duration of perceived intergoal relationships.

### Inter-rater reliability

Three independent researchers double-coded the transcripts to assess the inter-rater reliability of coding for control beliefs, goal interference, and goal facilitation. Each double-coder was assigned a random sample of interview transcripts along with instruction materials and practice coding. We used an iterative double-coding procedure. In step one, JP developed instruction materials and a practice sheet that an independent coder then used to code a random set of three interview transcripts. Coding results were compared and discussed in depth throughout this step of the double-coding procedure to clarify ambiguities or difficulties in the coding material instructions. Inter-rater reliability indices were not calculated at this step, given the extent of discussion between the coder and JP. In step two, we aimed to refine the instruction materials. A second coder was presented with the modified instruction materials and independently coded another random sample of four transcripts (overlap between coders one and two on one transcript). The coder and JP then compared coding and discussed discrepancies until a consensus was reached. Ambiguities in the instructions were discussed to further clarify the materials for the final double-coder. Inter-rater reliability at step two was tested using Krippendorff's alpha [41] over all constructs was α = 0.72 (95%CI 0.58 to 0.84). In step three, we conducted a final double-coding using the refined instructions. A third independent coder was provided with another random set of four transcripts to code from the remaining transcripts not yet double-coded, along with the finalised instructions. Discrepancies were discussed until a consensus was reached. In this final step, all constructs met the criterion for acceptable inter-rater reliability of Krippendorf's α = 0.80 [42]. Over all three constructs, α = 0.84 (95%CI 0.68 to 0.96). For control beliefs, α = 0.86 (95%CI 0.68 to 1.00), for goal interference, α = 0.85 (95%CI 0.39 to 1.00) and for goal facilitation, α = 0.82 (95%CI 0.64 to 0.96).

### Construct-specific coding

#### Control beliefs

Control beliefs were identified as any belief about factors or circumstances reported to make it easier, or difficult or impossible for GPs to perform the focal prescribing and advising behaviours. This was explicitly distinguished from behavioural beliefs, which focus on beliefs about the consequences of the behaviour, and normative beliefs, which focus on beliefs about which important other individuals or groups might approve of performing the behaviour or not [39].

#### Intention and past behaviour

We coded the strength of the GP's intention and the proportion of their next five patients with whom they intended to perform each focal behaviour, as well as the number of their last five patients with whom GPs self-reported performing each focal behaviour. We considered attributions for why GPs did not pursue each focal goal with all of the last five patients, or intended to with all of their next five patients, as potential control beliefs, behavioural beliefs, or normative beliefs, as well as potential sources of goal interference or goal facilitation.

#### Goal facilitation and goal interference

We identified and coded all the goals and behaviours that GPs reported as facilitating and/or interfering with performing the two target behaviours. Both explicit and coder-inferred goal interference and facilitation were coded. Goal facilitation was defined as any behaviour performed or goal pursued by the GP which either helpfully led to or had overlapping attainment strategies with the two target behaviours. Goal interference was defined as any behaviour performed or goal pursued by the GP that hindered or made it less likely that they would perform the two target behaviours.

## Results

### Participants

The 12 participating GPs' ages ranged from 29 to 50 years (mean = 40.3 years), and five were women. One-half of GPs had an affiliation with a university, and one-half practised in a rural setting. Graduation year ranged from 1981 to 2003 (median = 1989.5). GP contract (QOF) data from 2007/2008 for the percentage of patients with diabetes reaching a BP target of ≤145/85 mmHg indicated that participants' practices achieved this target with 75.60% (range greater than 20%) of their patients [33]. Six GPs reported aiming for a BP guideline target of ≤140/80 mmHg, four reported aiming for the GP contract (QOF) target of ≤145/85 mmHg, two reported aiming for a range rather than a specific target, and one GP reported prescribing until the patient no longer took the medication or had side effects. There was thus adequate heterogeneity on the key sample characteristics.

### Past behaviour

There was considerable variation in GP's self-reported provision of physical activity (PA) advice with their last five patients with diabetes with persistent hypertension, ranging from 'Probably none' (ID1, male, 43, rural) to 'at least three out of five I would say' (ID5, male, 41, urban) through to 'I would say all of them actually, in different degrees' (ID4, female, 34, rural). GPs reported providing PA advice to a median of two out of their last five patients with diabetes with persistent hypertension (range 0 to 5 patients). GPs reported prescribing to reduce BP with a median of 2.25 of their last five patients (range 0 to 4). Reports ranged from 'I think the last five patients, probably none actually' (ID3, female, 29, rural), through to 'I would say about four out of five' (ID11, male, 50, urban).

### Intention

Strength of intention to provide PA advice ranged between GPs from strong 'I think it's quite a strong intention' (ID2, female, 35, urban), 'it's relatively strong' (ID10, male, 47, rural) to weak 'fairly low I think, fairly low'(ID11, male, 50, urban). GPs reported intending to give PA advice to a median of 2.5 out of their next five patients (range 1.5 to 4), though one GP said 'almost none' (ID1, male, 43, rural) and another indicated 'if they are all overweight I would say it to all of them' (ID12, female, 30, rural). Strength of intention to prescribe to reduce BP was generally strong, but also depended upon other factors: 'so your intention is quite strong but there are so many other things that have to come into play' (ID2, female, 35, urban), 'well, just that you would [intend]. ... I definitely couldn't blanket say what I do with a group of patients as a whole' (ID3, female, 29, rural), 'well it depends on the class of drug they are already on' (ID9, male, 42, urban). GPs reported intending to prescribe to a median of 4.5 of their next five patients (range = 1 to 5). One GP mentioned 'I think that's very difficult to say because it's totally on an individual basis' (ID12, female, 34, rural).

### Control beliefs

We grouped control-related factors that GPs reported as making it easier or difficult for them to provide PA advice and prescribe to reduce BP into categories representative of similar content (See Additional File 2). All 12 GPs mentioned at least one control belief. Most reported that consultation factors and in particular that time-related pressures (mentioned by eight GPs) impeded their control over providing physical activity advice. For prescribing to reduce BP however, time pressures were highlighted by only three GPs. Most GPs reported that patient factors, namely patient preference for not wanting medication (mentioned by eight GPs), made it difficult for them to prescribe. We coded these as control beliefs because GPs believed that the patients' behaviour during the consultation affected their opportunity to perform their consistently strongly intended prescribing behaviour. This decision was made on the basis of Ajzen's definition of control beliefs, which suggests that it is a belief that 'deals with the presence or absence of requisite resources and opportunities' [4]. Had this been a subjective norm influence, the observed strong intention would not be expected. Thus, we viewed GPs' report of 'patient preference for not wanting a prescription' as a behaviour that the patient performs during the consultation that the GP believes affects their opportunity to prescribe in the consultation--*i.e.*, a control belief. One-half reported that patient factors (*i.e.*, patient interest and patient triggering the GP) made it easier for them to provide physical activity advice, though consultation factors (in particular 'having time'--three GPs) were also mentioned. For prescribing to reduce BP, patient factors were described as making it easier for GPs to prescribe, and in particular whether the patient is informed/understands the importance of BP in their diabetes management (five GPs). Consultation factors such as having time to discuss BP (three GPs) and having continuity of care (three GPs) were also seen as making it easier to prescribe. Overall, while GPs had relatively higher intention to prescribe than to give advice, BP prescribing was associated with more control beliefs.

### Goal interference

Table 1 shows that ten participants mentioned goal-directed behaviours that they perceived as interfering with providing PA advice, and seven GPs mentioned goal-directed behaviours perceived to interfere with prescribing to reduce BP. The majority of coded goal interference was elicited beyond control belief-related questions (92% of codes for BP prescribing and 82% for PA advice). Three participants mentioned that pursuing contract targets (*i.e.*, related to the GP contract) interfered with providing PA advice: 'it's the danger of targets and that they focus you on the targets which is their point, but it focuses you away from the non-targeted activities' (ID11, male, 50, urban). More GPs perceived that goal-directed behaviours related to the consultation in general interfered with providing PA advice than prescribing to reduce BP. Furthermore, the goal-directed behaviours perceived to interfere with PA advice had an enduring quality, *e.g.*, other priorities 'I think it's been squeezed out by everything else' (ID1, male, 43, rural); 'the nature of the beast is that I've got three things to cover here that need to be covered, and it takes less priority' (ID2, female, 35, urban). Conversely, the consultation goal-directed behaviours perceived as interfering with prescribing to reduce BP were more transient, *e.g.*, 'we need to capture a certain core of information for contract, so if it was kind of much last time we're going to see this patient this year, we've got to do blood screening, and BP treatment would probably be deferred until April or May' (ID6, male, 35, urban); 'I think the last five patients, probably none of them actually because I think it's all been patients with colds or I've seen them as a one-off' (ID3, female, 29, rural).

**Table 1:** Goal-directed behaviours perceived to interfere with focal behaviours during a consultation

Physical activity advice (N = 10 GPs)		Blood pressure prescribing (N = 7 GPs)	
**Theme**	**Goal-directed behaviours**	**Theme**	**Goal-directed behaviours**

Consultation (n = 8)	- fitting the patient agenda	Consultation (n = 4)	- capturing other GP contract information
	- focusing on GP contract-specific goals		- dealing with pressing issues
	- treating acute illness		- pursuing the contract BP targets
	- other clinical aspects (general)		- too much else going on in the consultation
		
Diabetes (n = 4)	- addressing medication		- treating acute illness
		
	- covering blood pressure and cholesterol	Diabetes (n = 2)	- addressing cholesterol
	- giving instruction for diabetic control		- multiple drugs to prescribe
	- getting HbA1c down		- talking about glycemic control
		
	- looking at blood sugar	GP/patient relationship (n = 3)	- providing patient choice
		
GP factors (n = 2)	- not wanting to be a broken record		- respecting patient preference
	- wanting to go home		

Participants perceived goal-directed behaviours specifically related to diabetes as interfering with both target behaviours, though more participants mentioned this as an issue for providing PA advice. While idiosyncratic, the goals of 'not wanting to be a broken record' (ID5, male, 41, urban) and 'wanting to go home in time for dinner' (ID1, male, 43, rural) highlight that GPs' personal goals can also potentially interfere with providing PA advice in the consultation.

### Goal facilitation

Table 2 shows that eleven of twelve participants mentioned goal-directed behaviours perceived to facilitate providing PA advice and prescribing to reduce BP in a consultation. Most coded goal facilitation was elicited beyond control belief related questions (71% of codes for BP prescribing and 79% of codes for PA advice). The focal behaviours were mentioned by participants as facilitating each other to a certain extent: 'it's difficult to just look at BP without looking at physical activity, these sorts of things [happen] at the same time' (ID3, female, 29, rural). Providing PA advice was perceived to be facilitated by discussing other lifestyle issues (particularly, 'weight discussions' was mentioned by seven of 11 GPs) and addressing diabetes-related risks for future health. Prescribing to reduce BP was perceived to be mainly facilitated by performing ongoing consultation goal-directed behaviours (*e.g.*, clearly structuring the consultation, trying to reach QOF targets, negotiating with the patient).

**Table 2:** Goal-directed behaviours perceived to facilitate focal behaviours during a consultation

Physical activity advice (N = 11)		Blood pressure prescribing (N = 11)	
**Theme**	**Goal-directed behaviour**	**Theme**	**Goal-directed behaviour**

Consultation (n = 1)	- Taking a history	Consultation (n = 6)	- Clearly structuring the consultation
		
Diabetes (n = 10)	- Addressing blood pressure		- Discussing diabetes as a whole
	- Addressing cholesterol		- Engaging the patient
	- Addressing HbA1c		- Negotiating with the patient
	- Discussing cardiovascular risk		- Advise patient to return if side effects
	- Discussing sugar control		- Trying to reach GP contract targets
		
	- Discussing heart and kidney risks	Discussion about future health (n = 5)	- Addressing HbA1C
		
Lifestyle (n = 8)	- Addressing alcohol		- Addressing poor sugar control
	- Addressing smoking		- Discussing cholesterol
	- Asking about work		- Discussing reducing risks
	- Checking BMI		- Showing CV risk
		
	- Checking general fitness	Lifestyle (n = 3)	- Exercise advice
	- Talking about weight		- Taking a holistic approach
	- Talking about diet		- Giving weight advice
		
	- Weighing the patient	Educating patient (n = 4)	- Re: medication and side effects
		
Mental health (n = 2)	- Addressing well-being		- Re: high blood pressure
	- Asking about low mood		- In general
	- Asking about stress		- Quoting guidelines
			- Showing results
		
		Prescribing (n = 3)	- Choosing drugs with good side effects
			- Explaining options
			- Following guidelines
			- Planning prescribing options

### Prospective goal facilitation

While this study focused on facilitating goal-directed behaviours within a specific consultation, participants also described goal-directed behaviours that prospectively facilitated performance of the focal behaviours. Nine of twelve GPs mentioned goal-directed behaviours that they performed over many consultations that eventually facilitated prescribing to reduce BP: building rapport, establishing shared or GP-led nature of consultation, giving opportunity to try lifestyle options first, recommending a home BP monitor, tailoring guidelines, using staged prescription of different drugs, providing written information, GP writing self reminders, inviting patients who are not at maximum tolerated dosage in for a review, and taking multiple BP readings. For providing physical activity advice, fewer GPs (four of twelve) mentioned comparatively less goal-directed behaviours that prospectively facilitated providing PA advice. These included empowering the patient, *e.g.*, 'really empowering the patient themselves to take a bit more responsibility for their own health and condition' (ID4, female, 34, rural), making another appointment with the GP, and making an appointment with the nurse.

## Discussion

### Main findings

This study used TPB-based constructs supplemented by a multiple goals approach to investigate control beliefs and the facilitating and interfering goal-directed behaviours that GPs perceived as affecting their performance of two evidence-based behaviours in a diabetes consultation. Results showed that indeed GPs perceived other goal-directed behaviours as interfering with and facilitating performing the focal evidence-based behaviours, though to a different extent between behaviours. The majority of perceived goal facilitation and interference was elicited beyond the standard control belief elicitation. Results were in line with quantitative research conducted with other populations that found that the interfering [18-20,43] and facilitating (Presseau J, Sniehotta FF, Francis JJ, Gebhardt WA: With a little help from my goals: Integrating intergoal facilitation with the theory of planned behaviour to predict physical activity, Submitted) [17] effect of other goal pursuits were related to the performance of a particular behaviour. This study contributes to this research by providing qualitative evidence that GPs perceive that goals they pursue when managing diabetes interfere with and facilitate their performance of evidence-based behaviours. This study adds to the literature by considering how both the content and duration of this perceived interference and facilitation may affect performance. In doing so, this study suggests promising lines of development of behavioural theory to reflect physicians' perceived competing demands in clinical practice. Behavioural approaches to implementation research may benefit from further investigation of the perceived influences of pursuing multiple goals over and above intentions and PBC.

### Content of perceived goal interference and facilitation between focal behaviours

While similar types of goal-directed behaviours were perceived to interfere with both focal behaviours (though more frequently for PA advice), BP prescribing was consistently described as strongly intended whereas intention to provide PA advice varied between GPs. This suggests an underlying (and perhaps not surprising) potential difference in relative priority between the two focal behaviours for some GPs. The implication is that when goals compete, the less prioritised goal-directed behaviours may be subject to a greater influence by other interfering goal-directed behaviours.

As opposed to goal interference, as many participants described goal-directed behaviours that facilitate giving PA advice as prescribing to reduce BP. Though some goal-directed behaviours were perceived to facilitate both focal behaviours (including each other), a key content-related difference distinguishes the two: one-half described 'consultation' goal-directed behaviours as facilitating BP prescribing (compared to one GP for PA advice), whereas eight described other 'lifestyle' goal-directed behaviours as facilitating giving PA advice (compared to three GPs for BP prescribing). Performing 'consultation' goal-directed behaviours may effectively provide a supportive context for performing the highly intended behaviour. Conversely, the behaviour with more variable levels of intention was not described as being facilitated by such consultation goal-directed behaviours, but rather by the cluster of other similar lifestyle goal-directed behaviours. These differences between focal behaviours again suggest an underlying difference in relative priority. When time is limited, we question whether facilitating similar (*e.g.*, other lifestyle) goal-directed behaviours would increase the likelihood of a focal behaviour being performed, because that facilitating effect would depend on those similar behaviours also being performed. However, facilitating goal-directed behaviours at the consultation level may provide a context that favours the facilitated focal behaviour despite time limitations. Certain types of goal-directed behaviours may therefore be more useful for promoting the performance of a focal evidence-based behaviour.

### Goal facilitation and interference along a temporal dimension

Despite the interviews focusing on perceived intergoal relations within a single consultation, the longitudinal and chronic nature of diabetes care was often reflected in GPs' responses when discussing facilitating goal-directed behaviours. This suggests that goal facilitation may operate beyond the single consultation and that pursuing such goals over a series of consultations eventually facilitates performing the focal behaviour (*i.e.*, prospective facilitation). While this lead-up prospective facilitation is reminiscent of Bandura's 'proximal subgoals' [44] and Bagozzi's 'instrumental acts' [45], the latter concepts are framed within a perspective that is explicitly focused on a single behaviour. Conversely, the concept of prospective goal facilitation takes a systems-based perspective. The system can be considered as made up of multiple goal-directed and valued behaviours that are performed in and of themselves, rather than expressly to facilitate a particular behaviour. This temporal perspective of prospective goal facilitation may help to account for the longitudinal aspects of general practice often recognised as a main advantage, such as continuity of care [46]. It also presents with the possibility of developing strategies for promoting facilitation based on planning (*e.g.*, facilitation planning) that extend over many consultations.

While an equivalent temporal dimension for goal interference was not overtly described by GPs, the perceived interfering relationship between goal-directed behaviours can nevertheless be considered along a temporal continuum. For instance, some identified interfering goal-directed behaviours can be considered as one-offs, representing a more transient form of interference confined to a single consultation (*e.g.*, treating an acute illness, dealing with pressing issues). Other goal-directed behaviours presented a more enduring interference because they are potentially performed frequently and recurrently over time (*e.g.*, fitting in the patient agenda, capturing other information for the GP contract). The advantage of distinguishing this temporal dimension lies in the possibility that separate strategies may exist for dealing with such perceived interfering goals. Transient interference can be dealt with using deferral strategies [47], whereas enduring interference is by definition longitudinal in nature and thus continuous deferral would likely be detrimental. Enduringly interfering goal pursuits may also be an indication of the relative priority of a goal-directed behaviour; if many goals interfere over a long period of time with performing a particular behaviour, the latter may not be seen as important or useful. Enduring interference may be particularly problematic for optimal performance of evidence-based behaviours, and future research could specifically identify whether duration of perceived interference affects performance of particular focal clinical behaviours. That said, identifying and promoting facilitating goal-directed behaviours may circumvent these more enduring perceived interfering goal-directed behaviours, as could re-evaluating, modifying or disengaging from a particularly interfering goal [47].

### Relative priority between goal-directed behaviours

The relative priority between the focal behaviours was an underlying finding in this study. Despite more barriers expressed for prescribing to reduce BP, it was also consistently described as strongly intended whereas intention to give PA advice was variable. Differences in relative priority are not surprising because PA advice can often also be provided by other primary care staff (*e.g.*, practice nurse), whereas prescribing to reduce BP is primarily the GP's role (though increasing dosage can be nurse-led). While some GPs may indeed prioritise diagnosing and treating diabetes, the variation in described strength of intention to give PA advice suggests that this is not true of all GPs. Future research should investigate whether perceptions about professional role influence the priority of a particular evidence-based clinical behaviour relative to other goal-directed behaviours performed in a consultation.

In a null-sum situation of limited time something must give way, and this is likely determined by the perceived priority of each goal-directed behaviour. However, applications of single behaviour models to health professional behaviour [5,8] inherently do not consider this. A GP may intend to address cholesterol and BP with a patient, and defer addressing BP to the next consultation in order to be able to pursue both. However, this still raises the question of which behaviour should take precedence and which should be deferred. This may be less of an issue when follow-up consultations or extra time slots [48] are readily available. However, the follow-up consultation also presents with another set of goal-directed behaviours themselves potentially interfering with the now deferred behaviour. Whether or not the deferred behaviour's priority has changed may again be a function of what other goal-directed behaviours the GP performs in the follow-up consultation. The effectiveness of strategies for dealing with interference and promoting facilitation may also ultimately depend on which goal-directed behaviours are prioritised at any given time. Given that BP prescribing for people with diabetes is currently related to a GP contract-remunerated target in the UK, while PA advice is not seems a likely reason for differences in relative priority. Indeed, relative priority is likely to be influenced by a number of behavioural, normative, and control beliefs, and future research focusing on influences of priority seems justified.

### Comparing control beliefs and perceived intergoal relationships

Control beliefs and perceived intergoal relationships have similarities; indeed both reflected similar themes in this study. In theory, one would expect intergoal conflict and facilitation to be reflected in perceptions of perceived control. Regardless of whether they represent a more detailed facet of control beliefs or are independent constructs, questions and prompts of goal facilitation and interference elicit content that might otherwise be missed in standard belief elicitation studies. Indeed, while some of the coded perceived intergoal relationships emerged following control belief elicitation, the vast majority of coded perceived goal facilitation and interference (71% to 92% of codes) was elicited using questions and prompts for these constructs or when discussing intention. In itself, this argues that it may be important to further consider the context within which focal clinical behaviours are performed, including competing goal-directed behaviours.

Further conceptual and empirical factors can also attest to their distinctiveness. Conceptually, control beliefs 'deal with the presence or absence of requisite resources and opportunities' [4]. Conversely, goal-directed behaviours compete for those resources and opportunities, are performed independently for their own sake, and are determined by their own set of beliefs, perceptions, and intentions. Perceived intergoal facilitation and interference are constructs that partly represent sources of resource competition, and thus may influence control beliefs about a particular goal-directed behaviour. For instance, 'focusing on GP contract goals' was described as a goal-directed behaviour that interfered with giving PA advice. Pursuing these perceived interfering contract goals may then lead the GP to perceive a time constraint (*i.e.*, a control belief). Perceived intergoal relationships might also influence other control-related beliefs. For instance, 'engaging the patient' and 'negotiating with the patient' were goal-directed behaviours described as facilitating prescribing to reduce BP, and their pursuit may influence control beliefs described as making it easier to prescribe, such as 'knowing the patient'. These examples suggest that perceived intergoal relationships may contribute towards control beliefs about a particular goal-directed behaviour, but are conceptually separate.

That said, despite our focus on control beliefs, perceived intergoal relationships may also inform other types of beliefs. For instance, the perceived facilitating effect of 'talking about weight' might affect a behavioural belief that it is good practice to talk about exercise, and the perceived interference of 'pursuing other GP contract targets' might affect normative beliefs about whether colleagues think the GP should prescribe. Furthermore, these perceived intergoal relationships may influence a behaviour without necessarily informing specific beliefs about the behaviour, leading to a independent influence on behaviour. While these effects require quantitative substantiation in a clinical sample, perceived intergoal facilitation has been shown to be partially mediated by the TPB and also additionally independently predict behaviour in a non-clinical population [Presseau J, Sniehotta FF, Francis JJ, Gebhardt WA: With a little help from my goals: Integrating intergoal facilitation with the theory of planned behaviour to predict physical activity, Submitted]. This further attests to the distinction between control factors and perceived intergoal relationships.

### Implications for implementation science

Implementation science is concerned with understanding and promoting the application of research into practice, which involves the behaviour of health professionals. Theory-based models of behaviour allow us to build a cumulative science to understand the factors that are perceived to relate to performing according to the standards set by current evidence. Investigations of extensions to such models of behaviour allow us to maintain their foundations while attempting to address identified shortcomings. This qualitative study contributes hypothesis-generating results towards the further development of behavioural theory to better understand such variations in evidence-based health professional behaviour. This study suggests that what GPs do and pursue during a consultation are perceived to influence each other in a helpful or hindering way. Rather than solely focusing on a single investigator-identified behaviour, busy time-constrained consultations may be more appropriately conceptualised by also explicitly considering the perceived influence of GPs' other goal-directed behaviours. Gaps between research evidence and the performance of a particular clinical behaviour might be addressed by focusing attention upon what else the GP wants to do and does during the consultation, and how they relate to the focal behaviour. In some instances, many of the other goal-directed behaviours in the consultation are perceived to interfere with its performance. For others, the extent of interference is lesser (perhaps due to a higher relative priority), though behaviour may still be marred by a number of identified control beliefs. The value of a multiple goal-directed behaviour approach to implementation science may be as a means of: assessing how higher-level policy driven goals such as 'provide patient centred care' and 'provide evidence-based care' are pursued (*i.e.*, goal-directed behaviours) and how these pursuits may facilitate or interfere with one another; identifying and promoting sustainable clinical goal pursuits that facilitate particular evidence-based behaviours; and identifying and addressing competing goal pursuits that interfere with these evidence-based behaviours.

For instance, eliciting the multiple goal-directed behaviours that professionals perform and assessing their perceived interfering and facilitating influence on a focal behaviour may raise the awareness and salience of otherwise habitually performed behaviours. This could provide the opportunity to target interfering goal relations (that may or may not be related to control belief-related barriers). Once this interference is identified, and if appropriate, strategies can be adopted to minimise its effects. In this study GPs reported that respecting patient choice interfered with prescribing to reduce BP (Table 1), and that whether the patient 'understands and is informed' made it easier to prescribe (Additional File 2). They also perceived that performing the goal-directed behaviour of 'educating patients' facilitated prescribing to reduce BP (Table 2). Thus, a strategy of educating patients may both facilitate performance of the target behaviour and promote the factors seen as making it easier to prescribe to reduce BP, minimising the potential influence of the interfering goal. Promoting such facilitating sequences of goal-directed behaviours uses the existing structure of goal pursuit, rather than necessarily introducing new goal-directed behaviours. This could involve prospective facilitation whereby facilitating goal-directed behaviours can be identified and prospectively planned to be performed over time, which may provide a theoretically-informed operationalisation of continuity of care.

### Strengths and limitations

This study used an explicit and *a priori*-specified theory-based methodology as a foundation for thematic analysis. This approach is a strength of this study because it allowed us to integrate knowledge and evidence from existing theories to extend current ones, rather than (re)inventing a new theory [49]. While further quantitative evidence is needed to substantiate the qualitative findings in this study, by moving beyond single behaviours studied in isolation, this study attempted to bring some clarity to the complexity of clinical practice. The theory-based methods support the results in contributing to building a cumulative evidence base of the implementation of health professional behaviour. Methodologically, the double coding and inter-rater reliability assessment are also a strength. While this study is limited by a small sample size, this is mitigated by the purposive heterogeneity sampling strategy used to explore the breadth of responses. It became evident in the later interviews that the research questions had been sufficiently answered, *i.e.*, that GPs did perceive their goal-directed behaviours as facilitating and influencing performing the two focal behaviours. Though the study was not designed to necessarily achieve data saturation, evidence from the literature suggesting that a sample size of 12 can provide as much information as a much larger sample in qualitative studies [37].

### Unanswered Questions

While the study design precludes us from drawing conclusions about whether perceived intergoal relationships might augment the TPB, this study nevertheless allows us to generate hypotheses, particularly when also considering research in non-clinical populations. Future investigations could test hypotheses regarding whether perceived intergoal relationships build independently on TPB constructs, or moderate the relationship between clinicians' intentions and their behaviour. Whether promoting facilitating goal pursuits and reducing the effect of interfering goals might affect performance of a focal behaviour also remains an open question. Another unanswered question involves GPs' reports of high intention to prescribe to reduce BP, but expressing conditions related to the situational demands of the consultation that affect that high intention; future research should consider the implications of these 'conditionalities'. Finally, it seems plausible to have a strong intention towards many behaviours while still prioritising some over others, as priority implies urgency. Future investigations distinguishing 'priority' from alternative comparative measures, such as intention-choice [11] or relative intention (*e.g.*, rank or difference between intention to perform multiple goal-directed behaviours) may contribute to understanding the effect that multiple goal-directed behaviours have on performing a focal behaviour.

## Summary

GPs perceive their other goal-directed behaviours as influencing the performance of particular focal behaviours. This hypothesis-generating result suggests that behavioural approaches to knowledge translation may benefit from further investigation of whether multiple goal-directed behaviour approaches can predict and explain variation in health professional behaviour beyond single-behaviour models.

## Ethical approval

Ethical approval for this study was obtained by the North of Scotland Research Ethics Committee.

## Competing interests

The authors declare that they have no competing interests.

## Authors' contributions

JP, FFS, JJF and NCC conceived and designed the study. JP carried out the interviews, conducted analyses and wrote the manuscript. All authors edited, revised and approved the final manuscript.

## Supplementary Material

Additional file 1 Interview topic guide. Click here for file

Additional file 2 Coded control beliefs for each focal behaviour (N = 12). Click here for file
