# Supplementary material for: Multiple goals and time constraints: perceived impact on physicians' performance of evidence-based behaviours
Source: Implement Sci. 2009 Nov 26;4:77. doi: 10.1186/1748-5908-4-77 (PMC2787492; doi:10.1186/1748-5908-4-77)
Supplement: Additional file 1 — Interview topic guide. [file 1748-5908-4-77-S1.DOC]

**Additional File 1. Interview Topic Guide**

**Preamble**

With this study, I want to try to get inside your head to try to understand how you deal with all the things that characterise a clinical consultation with your **adult** **patients who have Type 1 or Type 2 diabetes and have persistently high blood pressure as measured on at least the last three measurements**. I’m not a medic so my goal isn’t to make judgements about whether what you do is clinically ‘correct’. It might be useful to consider your patients as a whole, or as individual cases, and if it’s helpful, please feel free to confidentially consult any recent case notes you have.

Does that sound ok? Before we get started, do you have any initial comments or thoughts?

**Types of consultations**

I’d like to start by getting a sense of what sorts of consultations you have with patient of yours who have diabetes.

Q1: What types of consultations do you personally have?

Q2: How long do they typically last?

Q3: Which type are you personally most involved in?

**Elicitation of Goal-directed behaviours**

Q4: I’m interested in the activities and concerns that you have during a [insert Q3 consultation]. We call these your ‘personal projects’, so basically what you think about, plan for, carry out, and sometimes (though not necessarily always) complete. Some might be focused on achieving something with your patients or on the process of care; or things that you choose to do or feel you have to do; things you work towards or are trying to avoid. These personal projects can be related to any aspect of the consultation. What I’d like you to do is to think about [insert Q3 consultation] and describe it to me as the list of personal projects that you have.

- Are you able to get through this list with all your patients?
- What else would you pursue if you had more time in this type of consultation?

**Physical Activity – topic checklist**

- Providing PA advice something that you personally do in this type of consultation? In any type of consultation?
- How is PA advice given?
- Can you tell me about your intention to personally give PA-related advice during [insert Q3 consultation]? Can you briefly comment on the strength of your intention to personally give PA-related advice during [insert Q3 consultation]?
- Out of the next five patients with diabetes coming in for [insert Q3 consultation], to how many do you intend to personally give PA-related advice to? If not five, why not?
- What factors or circumstances enable you or make it easier to personally provide PA advice?
- What factors or circumstances make it difficult or impossible to personally provide PA advice?

- Thinking specifically about the list of personal projects that you have for a [insert Q3 consultation]

- Do any of the projects that you pursue in this type of consultation conflict in any way with giving physical activity-related advice? In what way?
- Does providing physical activity-related advice particularly conflict in any way with pursuing any other projects that you pursue? In what way?
- Do any of the projects that you mentioned that you pursue in this type of consultation in any way facilitate giving physical activity-related advice? In what way?
- Does providing physical activity-related advice in any way facilitate pursuing any other projects that you pursue? In what way?
- Any other issues that come to mind when you think about giving PA-advice during [insert Q3 consultation]?
- How many of your last five patients with diabetes did you personally give PA-related advice?

**Prescribing to reduce BP – topic checklist**

- In terms of reducing high BP, I know that some ways of doing that are to either add an additional BP-lowering drug or to increase the dosage of an existing drug.

- What is the target BP you aim towards for this type of patient (repeat the patient characteristics)?
- Is prescribing to reduce BP something that you do in this type of consultation? In any other type of consultation?

- I’d also like to talk a bit about the tighter recommendations such as reducing BP to <140/80?

- Can you tell me about your intention to either add an additional BP-lowering drug or to increase the dosage of an existing drug during [insert Q3 consultation]? Can you briefly comment on the strength of your intention? [both guideline and GP’s own target if different]
- Out of the next five patients with diabetes with high BP coming in for [insert Q3 consultation], for how many do you intend to either add an additional BP-lowering drug or increase the dosage of an existing drug until BP is <140/80? Until [their stated level if different]? If not five, why not? Would any of those people be exception reported?
- What factors or circumstances make it difficult or impossible? [both guideline and GP’s own target if different]. Anything else?
- What factors or circumstances enable you or make it easier? [both guideline and GP’s own target if different]. Anything else?

- Thinking specifically about your personal projects for a [insert Q3 consultation]

- Do any of the projects that you pursue in this type of consultation conflict in any way with prescribing until BP is <140/80? In what way?
- Does prescribing until BP is <140/80 conflict in any way with pursuing any of the other projects that you pursue? In what way?
- Do any of the projects that you mentioned that you pursue in this type of consultation in any way facilitate prescribing until BP is <140/80? In what way?
- Does prescribing until BP is <140/80 in any way facilitate pursuing any of the other things that you pursue? In what way?
- Any other issues that come to mind when you think about prescribing to reduce BP to <140/80 during an annual review follow-up?
- How many of your last five patients with diabetes did you either add an additional BP-lowering drug or increase the dosage of an existing drug until BP was <140/80? Until [their stated level if different]?

Thank you + Demographics
