# Supplementary material for: Multiple goals and time constraints: perceived impact on physicians' performance of evidence-based behaviours
Source: Implement Sci. 2009 Nov 26;4:77. doi: 10.1186/1748-5908-4-77 (PMC2787492; doi:10.1186/1748-5908-4-77)
Supplement: Additional file 2 — Coded control beliefs for each focal behaviour (N = 12). [file 1748-5908-4-77-S2.DOC]

| **Additional File 2. Coded control beliefs for each focal behaviour (N = 12)** | | |
| --- | --- | --- |
|  |  | |
| **Factors or circumstances that make it easier to…** | | |
| **…provide physical activity advice** | **...prescribe to reduce blood pressure** | |
| **Consultation factors (Nine GPs)** | **Consultation factors (Six GPs)** | |
| Having time | Knowing the patient | |
| Consultation related to exercise | Feedback from computer | |
| Low number of patients with diabetes | Having time to discuss | |
|  |  | |
| **GP factors (Five GPs)** | **GP factors (Three GPs)** | |
| Knowing the patient | GP communication skills | |
| GP knowing the evidence | GP learning or knowing evidence | |
| GP having a fit spouse |  | |
| GP taking a course | **Patient factors (Eight GPs)** | |
| GP active themselves | Patient is informed/understands | |
| Natural for the GP | Patient is keen | |
|  | Patient is ready | |
| **Patient factors (Six GPs)** | Patient trusts GP | |
| Patient interest | Patient turns up | |
| Patient as a trigger | Patient willing to take meds | |
|  |  | |
|  | **Environmental factors (Five GPs)** | |
|  | Good medication | |
|  | Guidelines | |
|  | Having leaflets and handouts | |
|  | Locality of consultation | |
| **Factors or circumstances that make it difficult to…** | | |
| **…provide physical activity advice** | | **...prescribe to reduce blood pressure** |
| **Consultation factors (Nine GPs)** | | **Consultation factors (Three GPs)** |
| Time constraints and pressures | | Time in consultation |
| Patient's agenda | | Patient agenda |
| Existence of contract targets | | Lot of work to persuade patient |
| People in waiting room | |  |
|  | | **Environmental factors (Four GPs)** |
| **Environmental factors (Two GPs)** | | BP drug effectiveness |
| Lack of practice's ability to prescribe PA | | Locality of consultation |
| Lack of sports facilities | | What patients read in the paper |
|  | | Hospital clinic |
| **GP factors (Two GPs)** | | Time for blood results to return |
| Believing patient reports of high PA levels | |  |
| GP's thoughts diverted | | **GP factors (Four GPs)** |
|  | | GP ability to persuade the patient |
|  | | GP attention |
|  | | GP familiarity with types of medication |
|  | |  |
|  | | **Patient factors (Nine GPs)** |
|  | | Patient avoiding follow-up |
|  | | Patient health beliefs |
|  | | Patient preference not to have (more) tablets |
|  | | Patient uses deferral tactic |
|  | | Patient wants to audit medication |
